# MAFLD and impaired bone formation in children: association with reduced serum LCAT

**DOI:** 10.3389/fcell.2026.1902360

**Published:** 2026-09-25

**Authors:** Lingyan Zhu, Lin Xu, Lanshu Yang, Baolan Sun, Weixia Yang

**Affiliations:** 1 Department of Pediatrics, Affiliated Hospital of Nantong University, Medical School of Nantong University, Nantong, Jiangsu, China; 2 Department of Pediatrics, Yingfeng Branch, General Hospital of the Medical Consortium of Zhejiang Xiaoshan Hospital, Hangzhou, Zhejiang, China; 3 Department of Pediatrics, Chongzhou Maternal and Child Healthcare Hospital, Chengdu, Sichuan, China; 4 Department of Laboratory, Affiliated Hospital of Nantong University, Nantong, Jiangsu, China

**Keywords:** pediatrics, obesity, MAFLD, bone metabolism, LCAT, liver-bone axis

## Abstract

**Purpose:**

To evaluate bone metabolism indicators and serum lecithin–cholesterol acyltransferase (LCAT) levels in children with metabolic dysfunction-associated fatty liver disease (MAFLD), and to explore their interrelationship.

**Patients and Methods:**

This study enrolled 127 children aged 6–14 years, classified into control (n = 42), obesity without hepatic steatosis (n = 41), and MAFLD (n = 44) groups. All participants underwent clinical evaluation, laboratory testing, and liver ultrasonography. Serum LCAT was quantified by enzyme-linked immunosorbent assay (ELISA). Bone turnover markers, including procollagen type I N-terminal propeptide (PINP), β-C-terminal telopeptide of type I collagen (β-CTX), and 25-hydroxyvitamin D [25-(OH) D], were measured via electrochemiluminescence. Independent risk factors for impaired bone formation were analyzed using multivariable ordinal logistic regression.

**Results:**

Serum LCAT concentrations were highest in controls (58.27 ± 4.43 mg/L), followed by obesity without hepatic steatosis (46.13 ± 3.37 mg/L), and lowest in the MAFLD group (40.71 ± 3.44 mg/L) (*P* < 0.001). The proportion of children with PINP below the 25th percentile was significantly greater in the MAFLD group (88.64%) than in obesity (48.78%) and control groups (35.71%) (*P* < 0.001). Significant intergroup differences were observed for 25-(OH) D, PINP values, PINP grade, and PINP/β-CTX ratio; PINP grade distribution differed among groups (*P* < 0.001), whereas β-CTX grade did not. Spearman analysis revealed significant correlations of LCAT with HDL-C, AST, ALT, fatty liver status, 25-(OH) D, and PINP grade (all *P* < 0.001). Multivariable ordinal logistic regression identified MAFLD (*OR* = 4.74, 95% CI: 1.41–15.99, *P* = 0.012) and older age (*OR* = 1.38, 95% CI: 1.08–1.75, *P* = 0.009) as independent factors associated with lower PINP grade, whereas LCAT was not independently associated with PINP grade.

**Conclusion:**

MAFLD was independently associated with lower PINP status in children. Reduced serum LCAT levels were associated with MAFLD and unfavorable bone formation status but were not independently associated with PINP grade after multivariable adjustment. LCAT may therefore represent an associated biomarker within the liver–bone axis, requiring validation through prospective studies, formal mediation analyses, and mechanistic experiments.

## 1 Introduction

Metabolic dysfunction-associated fatty liver disease (MAFLD), formerly known as non-alcoholic fatty liver disease (NAFLD) (Eslam et al., 2021), has become the most common chronic liver disease in children and adolescents (Vimalesvaran and Dhawan, 2026). The overall prevalence of MAFLD in children is approximately 10%, which is closely related to multiple factors such as obesity, ethnicity, perinatal conditions, and genetics, posing a serious threat to long-term health (Schwimmer et al., 2025).

The liver serves as a central hub for systemic metabolism. The concept of the “Liver-Bone Axis” initially proposed by Nussler’s team in 2014, describes the bidirectional physiological regulation between the liver and bone through secreted factors and metabolites (Nussler et al., 2014). A healthy liver secretes protective factors such as insulin-like growth factor-1 (IGF-1) and lecithin–cholesterol acyltransferase (LCAT) to support bone health. In contrast, an impaired liver, as in MAFLD, may secrete detrimental factors like fibroblast growth factor 21 (FGF21) and insulin-like growth factor binding protein 1 (IGFBP1) or reduce the secretion of protective factors, thereby disrupting bone homeostasis and contributing to bone loss (Zhao et al., 2023). Several studies have described an association between MAFLD and disorders of bone metabolism (Drapkina et al., 2023).

LCAT, a key enzyme synthesized and secreted by the liver, circulates primarily in free form or bound to lipoproteins. It catalyzes the esterification of cholesterol into cholesteryl esters, facilitates high-density lipoprotein (HDL) maturation and reverse cholesterol transport (RCT), and promotes hepatic cholesterol clearance, playing a central role in lipid metabolism (Zanotti et al., 2022). Beyond its potential as a therapeutic target for atherosclerosis, emerging preclinical evidence suggests that LCAT may influence bone metabolism by mediating cholesterol efflux in bone tissue (Pownall et al., 2021). A preliminary study has found that a bone-targeted liposomal strategy can enhance hepatic LCAT secretion and improve bone remodeling by promoting bone formation and suppressing bone resorption in an experimental osteoporosis model, supporting a potential functional role of LCAT in the liver-bone axis (You et al., 2025). Furthermore, Lu et al. demonstrated that inhibition of PP2Acα increased hepatic LCAT expression and enhanced reverse cholesterol transport, thereby alleviating hepatic fibrosis and improving bone density in mice (Lu et al., 2022).

A primary study indicates that school-aged children with MAFLD have significantly lower bone density than their peers without MAFLD (Liu et al., 2026), further underscoring its detrimental effect on bone health. Childhood and adolescence are critical periods for bone remodeling, during which the rate of bone formation significantly exceeds that of bone resorption, leading to the accumulation of peak bone mass (PBM) (Pageau et al., 2025). Internationally, procollagen type I N-terminal propeptide (PINP) and C-terminal telopeptide of type I collagen (β-CTX) are recommended as reference markers for evaluating bone formation and resorption, respectively (Bhattoa et al., 2025). PINP and β-CTX are biochemical metabolites reflecting osteoblast and osteoclast activity. As sensitive serum markers of bone turnover, their changes often precede alterations in bone mineral density (Williams et al., 2024; Eastell et al., 2025). Their levels are influenced by geographical, lifestyle, demographic, and developmental factors (Motooka and Matsuo, 2020; Rand et al., 2023; Siderius et al., 2023). Vitamin D, primarily measured as serum 25-hydroxyvitamin D [25-(OH) D], also plays a crucial role in calcium and phosphorus homeostasis and is commonly used to assess nutritional status (Ogorek and Samara, 2025).

To date, no studies have examined serum LCAT levels in children with MAFLD, and it remains unclear whether LCAT is related to impaired bone metabolism in this population. This study aims to measure serum LCAT and bone metabolism markers in children and, after adjusting for confounding factors such as obesity, to explore whether LCAT plays a role in the liver-bone axis in pediatric MAFLD.

## 2 Materials and methods

### 2.1 Study population

This study recruited children from the pediatric endocrinology outpatient and inpatient departments of the Affiliated Hospital of Nantong University between January 2024 and September 2024. Through a stepwise screening process, 127 children were enrolled and categorized into three groups: 42 control children, 41 with obesity-without-hepatic-steatosis, and 44 with MAFLD (Figure 1). The three groups did not differ significantly in chronological age. The study complied with the Declaration of Helsinki and was approved by the Human Research Ethics Committee of the Affiliated Hospital of Nantong University (approval no.: 2024-K140-01). Written informed consent was obtained from all participants and their parents or guardians.

**FIGURE 1:**
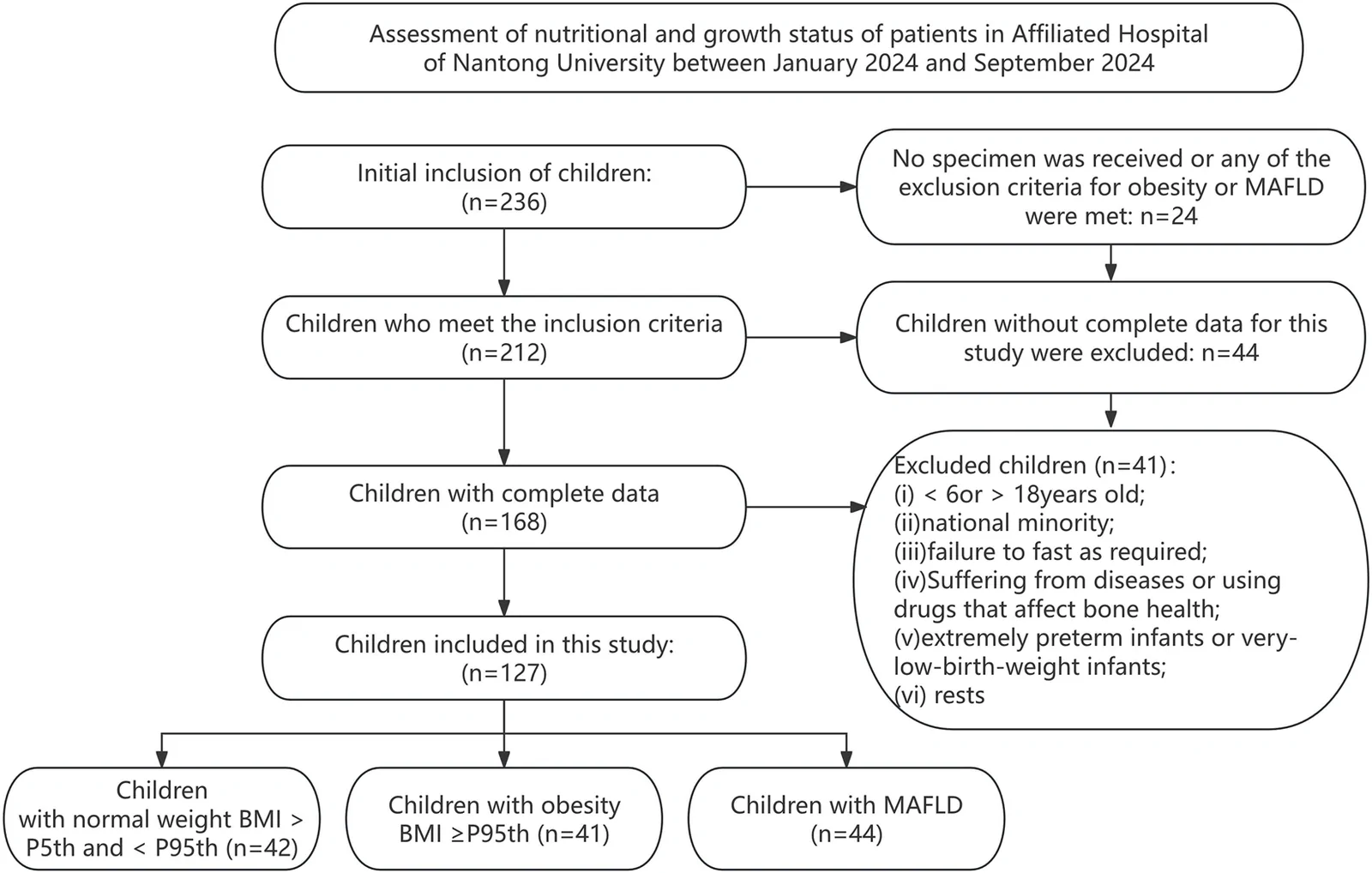
The specific screening process of the research subjects.

The evaluation indicators include the following sections: Measure the height and weight of children using standardized instruments, and calculate the BMI (weight (kg)/height (m)^2^). Pubertal developmental stages were assessed by pediatric endocrinologists using the Tanner staging system and categorized as follows: (1) Prepubescent: No signs of pubertal development in both sexes. (2) Early to mid-puberty: Girls at breast stages II–III; boys with testicular volumes of 4–15 mL. (3) Late puberty/post-pubertal: Girls at breast stages IV–V or post-menarche; boys with testicular volume ≥15 mL or presence of sperm (Wu et al., 2024).

The inclusion criteria were as follows: (1) obesity, defined as the BMI greater than the 95th percentile for children of the same age and sex (Jebeile et al., 2022); (2) age between 6 and 14 years; and (3) availability of liver ultrasound scan measurements. The exclusion criteria were as follows: (1) liver or kidney dysfunction due to conditions other than MAFLD; (2) severe hepatic or renal insufficiency; (3) a history of alcohol consumption or malignancy; (4) receipt of hormone therapy within the previous 3 months; and (5) any condition potentially affecting bone metabolism. The latter category included extreme prematurity, extremely low birth weight, disorders of pubertal development, and endocrine dysfunction (e.g., of the parathyroid, thyroid, or growth hormone axes), precocious puberty, primary childhood osteoporosis, any chronic systemic disease, a family history of significant bone disease, intake of food prior to blood sampling, or ongoing treatments known to influence bone metabolism.

The diagnosis of hepatic steatosis was based on established sonographic criteria, including hepatorenal echo contrast, parenchymal brightness, deep attenuation of the ultrasound beam, and vessel blurring (Chan et al., 2026). As all children in the experimental group were obese, the diagnostic criteria for MAFLD followed current international consensus guidelines (Eslam et al., 2021). Accordingly, obese children with ultrasound-confirmed hepatic steatosis were classified as having MAFLD. Children with a BMI above the 95th percentile for age and sex and without ultrasonographic evidence of hepatic steatosis were classified as the obesity-without-hepatic-steatosis group. Children with normal BMI and no fatty liver served as the control group.

### 2.2 Collection of clinical data and calculation of relevant indices

All clinical information of the children was collected and analyzed for gender, age, body mass index (BMI), puberty stage, fasting blood glucose (FBG), fasting insulin (FINS), C-peptide, triglycerides (TG), total cholesterol (TC), low density lipoprotein (LDL-C), high density lipoprotein (HDL-C), alanine aminotransferase (ALT), aspartate aminotransferase (AST), γ-glutamyl transferase (γ-GGT), Lactic dehydrogenase (LDH), alkaline phosphatase (ALP), and uric acid (UA). HOMA-IR = FPG (mmol/L) × FINS (mIU/L)/22.5.

### 2.3 Collection and processing of blood specimens

Venous blood samples were generally collected in the early morning after an overnight fast of at least 10 h. The exact clock time of sampling was not prospectively recorded. An additional 5 mL of fasting venous blood was collected and centrifuged at 3,500 rpm for 10 min. We then aspirated the serum, divided it into 2 EP tubes, sealed them and labeled the corresponding information of the children. The samples were stored in a −80 °C refrigerator to avoid repeated freezing and thawing.

Serum LCAT concentrations were measured using a commercial ELISA kit (AMEKO, Shanghai Lianshuo Biological Technology Co., Ltd., Shanghai, China; AE98684Hu). The manufacturer-specified detection range was 10–350 ng/L, with intra- and inter-assay CVs of <9% and <15%, respectively. Samples were measured after high-fold dilution, and the resulting concentrations were corrected for the actual dilution factor and converted to mg/L for statistical analysis.

Serum total PINP, β-CTX, and 25-(OH)D were measured by electrochemiluminescence immunoassay on a cobas 8,000 e602 analyzer (Roche Diagnostics). Total PINP was measured using the Elecsys total P1NP assay (REF 03141071190), with a measuring range of 5–1,200 ng/mL and manufacturer-reported repeatability and intermediate-precision CVs of 1.7%–3.2% and 2.3%–3.7%, respectively. 25-(OH)D was measured using the Elecsys Vitamin D total assay (REF 05894913190), with a measuring range of 3.00–70.0 ng/mL, a detection limit of 3.00 ng/mL, and a quantification limit of 5.00 ng/mL. Roche ECLIA reagents were stored at 2–8 °C and were not frozen.

### 2.4 The secondary conversion of PINP and β-CTX values

To ensure appropriate reference standards, this study adopted age-, sex-, and puberty-specific BTM reference curves established by Wu Chushan et al. in the Shenzhen region of China (Wu et al., 2024). Measured PINP and β-CTX values were converted into percentiles (PINP grade and β-CTX grade) and categorized into four intervals: >P75, P50–P75, P25–P50, and <P25.

### 2.5 Statistical analysis

Statistical analyses were performed using SPSS (version 27.0) and the Storm Statistical Platform. Normally distributed quantitative data are presented as mean ± standard deviation, while non-normally distributed data are summarized as median (P25, P75). Categorical variables are expressed as numbers and percentages (%). For intergroup comparisons, one-way ANOVA was applied for normally distributed data, and the Kruskal–Wallis test was used for non-normally distributed data. Differences in categorical variables were assessed using the chi-square test. Spearman’s rank correlation was employed for correlation analysis. To further examine the relationship between serum LCAT and PINP grade, partial Spearman correlation analyses were performed with sequential adjustment for age and BMI, followed by additional adjustment for MAFLD status. To evaluate factors associated with bone formation status, PINP percentile grades were coded ordinally (>P75 = 0, P50-P75 = 1, P25-P50 = 2, <P25 = 3) and analyzed using univariate and multivariable ordinal logistic regression. The primary multivariable model was kept parsimonious in view of the modest sample size and the sparse distribution of PINP categories. Because PINP percentile categories were derived from age-, sex-, and puberty-specific reference curves, sex and pubertal stage were already incorporated into the standardization of the outcome. Chronological age was nevertheless retained to account for possible residual age-related variation in skeletal maturation. Additional covariate-adjustment sensitivity analyses were performed based on Model 2, which included MAFLD status, age, BMI, and LCAT. Model A additionally included sex and Tanner stage; Model B additionally included 25-(OH)D and HOMA-IR; and the fully adjusted model included all four additional covariates. Detailed results are provided in Supplementary Table S1. The proportional-odds assumption for the primary ordinal logistic regression models was evaluated using the Brant test, including both omnibus and variable-specific tests. A nonsignificant result was interpreted as indicating no evidence against the proportional-odds assumption. If the assumption had been violated for individual covariates, a partial proportional-odds model would have been considered as an alternative approach. Multicollinearity among predictors included in the multivariable model was assessed using variance inflation factors (VIFs), tolerance values, and the condition number. VIF values < 5 and tolerance values > 0.20 were considered to indicate the absence of problematic multicollinearity. Given the sparse distribution of PINP categories, additional sensitivity analyses were performed to assess the robustness of the primary findings. A mean bias-reduced adjacent-category ordinal regression model was fitted while retaining all four PINP categories. In addition, PINP status was dichotomized as < P25 versus ≥ P25 and analyzed using Firth penalized logistic regression. Both sensitivity models included MAFLD status, age, BMI, and LCAT as covariates. Because several expected cell counts in the 3 × 4 group-by-PINP-grade contingency table were small, the overall difference in PINP-grade distribution among the three groups was further evaluated using the Monte Carlo Fisher-Freeman-Halton exact test with 1,000,000 replicates. Because no formal *a priori* sample-size calculation was performed, sensitivity power analyses were conducted using the available sample. For the 3 × 4 comparison of PINP-grade distributions, Cohen’s ω was calculated as the overall effect size, and the minimum detectable effect size was estimated for N = 127, α = 0.05, and 80% power. For the multivariable ordinal regression, a fixed-design simulation-based power analysis was performed using the fitted Model 2. The observed covariate matrix was retained, ordinal PINP outcomes were generated under the fitted proportional-odds model, and the same model was refitted across 5,000 simulated datasets. Power was estimated as the proportion of simulations in which the adjusted MAFLD effect reached two-sided statistical significance at *P* < 0.05. A two-sided p-value of less than 0.05 was considered statistically significant.

## 3 Results

### 3.1 Participant characteristics

There was no significant difference in the average age of the three groups of participants, and they were comparable: (11.17 ± 1.72) years in the MAFLD group, (10.44 ± 1.71) years in the obesity group, and (10.47 ± 1.52) years in the control group (*P* = 0.073). There were also no significant differences in the distribution of gender and puberty stage (*P* = 0.138; *P* = 0.988) (Table 1).

**TABLE 1:** Basic characteristics and differential analysis.

Indicators	Control group (n = 42)	Obesity without hepatic steatosis group (n = 41)	MAFLD group (n = 44)	P-value
Age (years)	10.47 ± 1.52	10.44 ± 1.71	11.17 ± 1.72	0.073
Gender (M/F)	18/24	20/21	28/16	0.138
Puberty stage (1/2/3)	17/19/6	16/19/6	17/19/8	0.988
BMI (kg/m^2^)	16.68 (15.79.18.54)	25.04 (24.26.27.15)^a^	29.10 (27.21.31.70)^ab^	<0.001
Fatty liver	0	0	44	<0.001
FINS (mIU/L)	8.36 (5.77.11.90)	13.25 (9.72.17.26)^a^	26.19 (18.23.40.20)^ab^	<0.001
FBG (mmol/L)	4.30 (4.10, 4.80)	4.80 (4.50.5.10)^a^	5.00 (4.68.5.20)^a^	<0.001
HOMA-IR	1.67 (1.12, 2.30)	3.09 (2.16.3.91)^a^	5.78 (3.87.8.32)^ab^	<0.001
C-peptide (ug/L)	1.91 (1.57, 2.20)	2.19 (1.98.2.96)^a^	3.95 (3.05.4.79)^ab^	<0.001
TC (mmol/L)	4.10 (3.80.4.50)	4.10 (3.60.4.50)	4.70 (3.98.5.03)^ab^	0.016
TG (mmol/L)	0.99 ± 0.39	1.11 ± 0.35	1.58 ± 0.63^ab^	<0.001
LDL-C (mmol/L)	2.79 ± 0.41	2.56 ± 0.61	2.88 ± 0.73^b^	0.048
HDL-C (mmol/L)	1.38 (1.25, 1.55)	1.30 (1.18.1.42)	1.20 (1.10.1.28)^ab^	<0.001
AST (U/L)	20.00 (17.00, 23.00)	24.00 (21.00.31.00)^a^	35.50 (25.00.47.25)^ab^	<0.001
ALT (U/L)	25.00 (22.00, 29.75)	24.00 (20.00.33.00)	45.50 (27.50.76.00)^ab^	<0.001
γ-GGT (U/L)	16.00 (12.00, 18.00)	18.00 (16.00.23.00)^a^	27.00 (22.00.38.25)^ab^	<0.001
ALP (U/L)	342.02 ± 76.28	318.78 ± 68.89	284.89 ± 99.82^a^	0.007
LDH (U/L)	298.57 ± 72.80	278.63 ± 62.90	274.23 ± 38.21	0.137
AST/ALT	0.82 (0.73.0.89)	0.94 (0.76.1.19)^a^	0.75 (0.58.1.05)^b^	0.009
UA (μmol/L)	271.50 (232.50,320.25)	366.00 (336.00,424.00)^a^	405.00 (368.25,458.75)^a^	<0.001

a: *P* < 0.05 compared to the control group; b: *P* < 0.05 compared to the obesity without hepatic steatosis group; Abbreviations: ALP, alkaline phosphatase; ALT, alanine aminotransferase; AST, aspartate aminotransferase; BMI, body mass index; FBG, fasting blood glucose; FINS, fasting insulin; HDL-C, High-density lipoprotein; HOMA-IR, Homeostasis model-insulin resistance; LDH, lactic dehydrogenase; LDL-C, Low-density lipoprotein; TC, total cholesterol; TG, Trig1yceride; UA, uric acid; γ-GGT, γ-glutamyl transpeptidase.

Significant intergroup differences were found in multiple metabolic parameters. Post-hoc pairwise comparisons revealed that both the MAFLD and obesity without hepatic steatosis groups had significantly higher values for BMI, FINS, C-peptide, FBG, HOMA-IR, UA, AST, and γ-GGT compared to the control group (*P* < 0.05). Furthermore, the MAFLD group exhibited significantly elevated TC and ALT, along with lower ALP and HDL levels, relative to the control group (*P* < 0.05). When compared to the obesity without hepatic steatosis group, children with MAFLD showed significantly increased TG, TC, LDL, BMI, FINS, C-peptide, HOMA-IR, AST, ALT, and γ-GGT (*P* < 0.05), and significantly decreased AST/ALT ratio and HDL levels (*P* < 0.05).

### 3.2 Comparison of LCAT levels across groups

Serum LCAT concentrations differed significantly among the three groups (Figure 2). The highest level was observed in the control group (58.27 ± 4.43 mg/L), followed by the obesity without hepatic steatosis group (46.13 ± 3.37 mg/L), and the MAFLD group showed the lowest concentration (40.71 ± 3.44 mg/L). Post-hoc tests confirmed that LCAT levels in both the obesity without hepatic steatosis and MAFLD groups were significantly lower than those in the control group (*P* < 0.001). Furthermore, the MAFLD group had significantly lower LCAT levels than the obesity without hepatic steatosis group (*P* < 0.001).

**FIGURE 2:**
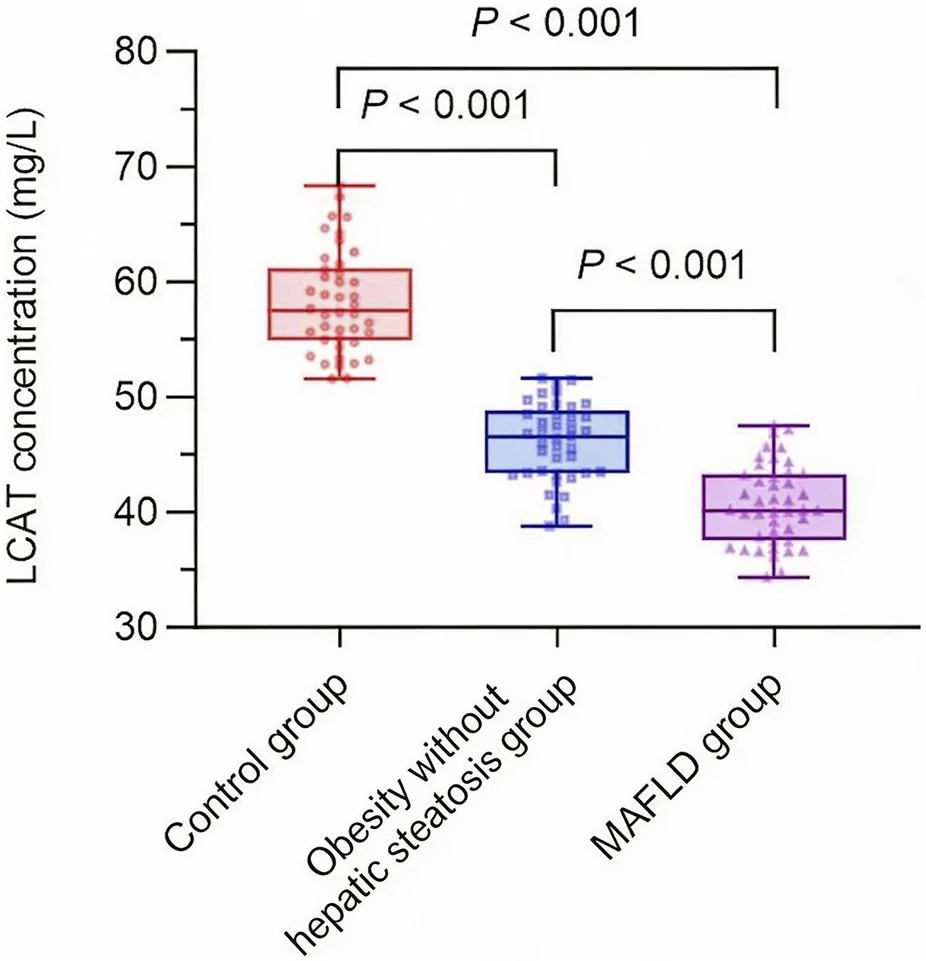
Scatter plot comparing LCAT levels among different groups. Data are presented as median (horizontal bar), 25th and 75th percentile (boxes), and 10th and 90th percentile (error bar).

### 3.3 Comparison of bone metabolism-related indexes across groups

Significant differences were observed among the three groups in serum 25-(OH)D concentrations, PINP concentrations, PINP grade distribution, and the PINP/β-CTX ratio (all overall *P* < 0.001; Table 2; Figure 3). As shown in Figures 3A–C, both the MAFLD and obesity-without-hepatic-steatosis groups had significantly lower serum 25-(OH)D concentrations, PINP concentrations, and PINP/β-CTX ratios than the control group. Compared with the obesity-without-hepatic-steatosis group, children with MAFLD had significantly lower PINP concentrations and PINP/β-CTX ratios (*P* < 0.05), whereas no significant difference in 25-(OH)D concentrations was observed between these two groups.

**TABLE 2:** Comparison of bone metabolism indexes among the three groups.

Indicators	Control group (n = 42)	Obesity without hepatic steatosis group (n = 41)	MAFLD group (n = 44)	P-value
PINP (ng/mL)	685.60 (573.73,800.40)	612.80 (491.10,718.50)^a^	492.95 (406.02,558.62)^ab^	<0.001
β-CTX (pg/mL)	988.70 (830.60,1193.00)	1,226.00 (990.10,1570.00)^a^	1,169.50 (965.68,1427.25)	0.052
25-(OH) D (ng/mL)	26.12 (19.72.32.29)	17.23 (14.02.22.66)^a^	16.20 (12.20.22.33)^a^	<0.001
PINP/β-CTX	0.68 (0.51.0.97)	0.49 (0.36.0.61)^a^	0.36 (0.29.0.53)^ab^	<0.001
PINP grade n (%)	​	​	​	<0.001
<P25	15 (35.71)	20 (48.78)	39 (88.64)	​
P25-P50	10 (23.81)	14 (34.15)	2 (4.55)	​
P50-P75	11 (26.19)	5 (12.20)	2 (4.55)	​
>P75	6 (14.29)	2 (4.88)	1 (2.27)	​
β-CTX grade n (%)	​	​	​	0.053
<P25	26 (61.90)	11 (26.83)	16 (36.36)	​
P25-P50	8 (19.05)	14 (34.15)	13 (29.55)	​
P50-P75	3 (7.14)	8 (19.51)	5 (11.36)	​
>P75	5 (11.90)	8 (19.51)	10 (22.73)	​

a: *P* < 0.05 compared to the control group; b: *P* < 0.05 compared to the obesity without hepatic steatosis group; Abbreviations: PINP, Procollagen type I N-terminal propeptide; β-CTX, C-terminal telopeptide of type I collagen.

**FIGURE 3:**
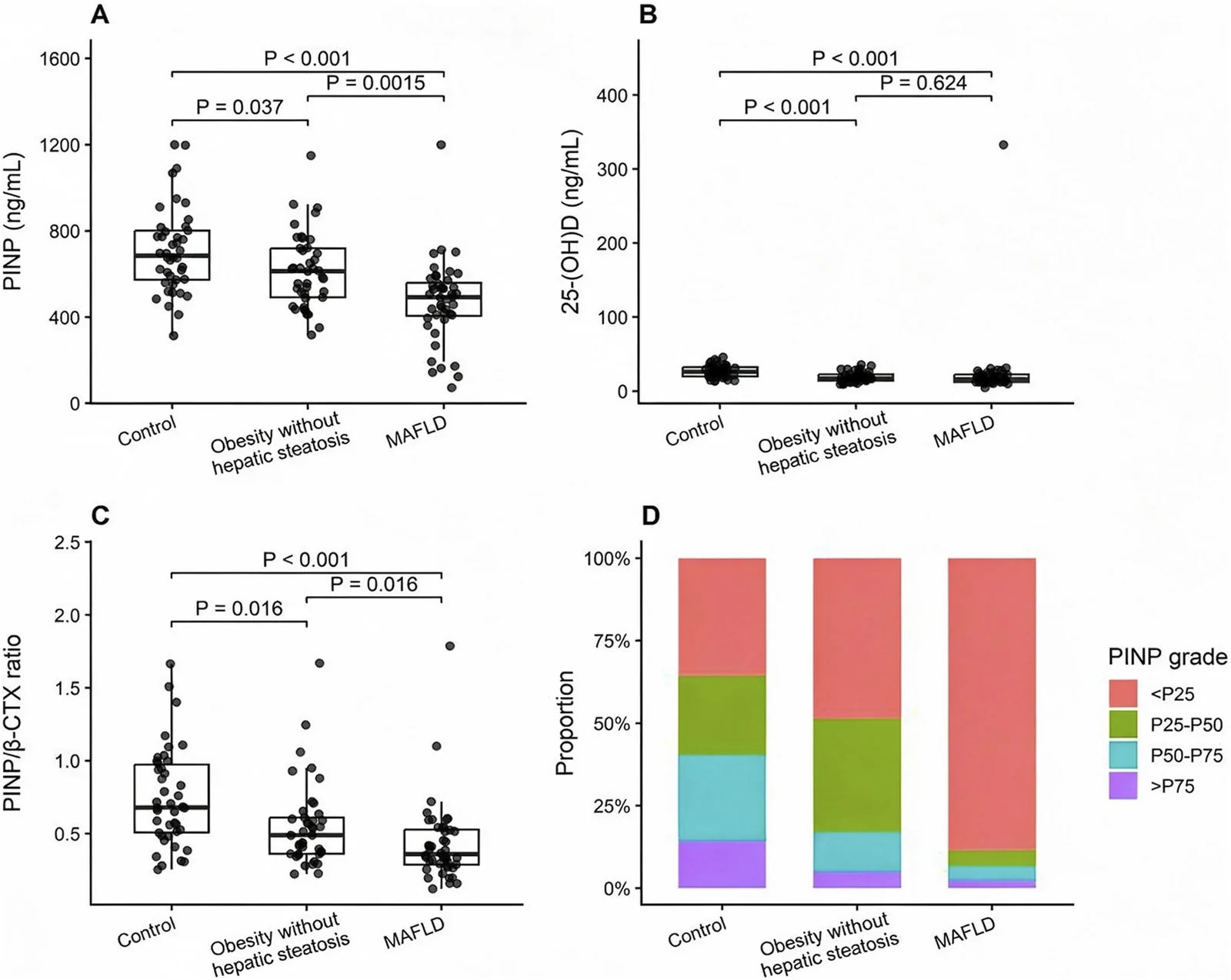
Distribution of bone metabolism-related variables showing significant differences among the three study groups. **(A)** Serum procollagen type I N-terminal propeptide (PINP) concentrations; **(B)** serum 25-hydroxyvitamin D [25-(OH)D] concentrations; **(C)** PINP/β-C-terminal telopeptide of type I collagen (β-CTX) ratio; and **(D)** distribution of PINP percentile grades. In **(A–C)**, individual dots represent individual participants, and box-and-whisker plots show the median and interquartile range. In panel D, proportional stacked bars show the within-group distributions of PINP grades (<P25, P25-P50, P50-P75, and >P75). The three study groups were classified as control, obesity without hepatic steatosis, and metabolic dysfunction-associated fatty liver disease (MAFLD).

The distribution of PINP grades also differed significantly among the three groups (*P* < 0.001; Figure 3D). Notably, the proportion of children with PINP below the 25th percentile (<P25) was substantially higher in the MAFLD group (88.64%) than in the obesity-without-hepatic-steatosis group (48.78%) and the control group (35.71%). In contrast, neither serum β-CTX concentrations nor β-CTX grade distributions differed significantly among the three groups (overall *P* = 0.052 and *P* = 0.053, respectively).

### 3.4 Correlation analysis of LCAT

Serum LCAT levels demonstrated a significant positive correlation with HDL-C (*r* = 0.357, *P* < 0.001) and 25-(OH) D (*r* = 0.327, *P* < 0.001), and were inversely correlated with AST (*r* = −0.512, *P* < 0.001), ALT (*r* = −0.307, *P* < 0.001), fatty liver status (*r* = −0.713, *P* < 0.001), and PINP grade (*r* = −0.403, *P* < 0.001) (Table 3; Figure 4).

**TABLE 3:** Spearman’s correlation analysis of each factor with LCAT.

Indicators	LCAT	
	*r*	*P*-value
HDL-C	0.357	<0.001
AST	−0.512	<0.001
ALT	−0.308	<0.001
Fatty liver	−0.713	<0.001
25-(OH) D	0.328	<0.001
PINP grade	−0.403	<0.001

**FIGURE 4:**
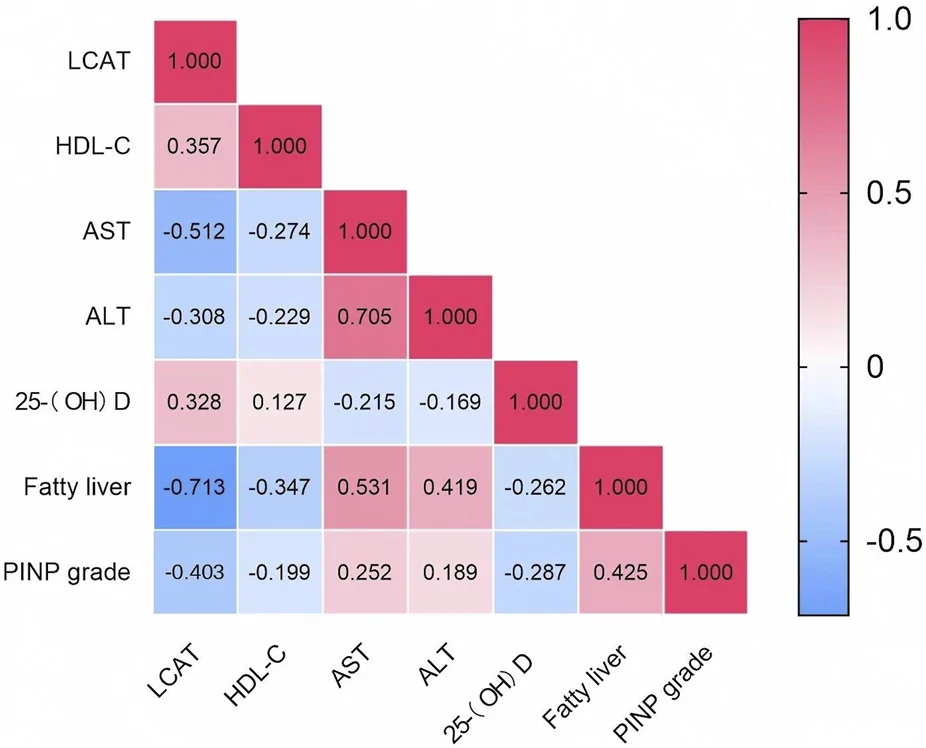
Spearman correlation analysis heatmap. Variables LCAT are correlated with variables HDL-C, AST, ALT, 25-(OH) D, Fatty liver, and PINP grade. The P values are all < 0.001. The results were transformed into a heatmap using the GraphPad Prism plotting tool.

In partial Spearman correlation analyses, the association between LCAT and PINP grade remained significant after adjustment for age and BMI (partial *r* = −0.222, *P* = 0.013), although the magnitude of the correlation was reduced. After further adjustment for MAFLD status, the association was further attenuated and was no longer statistically significant (partial *r* = −0.122, *P* = 0.177).

### 3.5 Regression analysis

Univariate ordinal logistic regression analysis was performed to identify factors associated with lower PINP grade (using > P75 as the reference). The results indicated that the presence of fatty liver, older age, higher BMI, FINS, C-peptide, TG, AST, ALT, γ-GGT, and HOMA-IR were significant risk factors for a decreased PINP grade (*OR* > 1, *P* < 0.05). In contrast, higher serum LCAT level was associated with a reduced risk of a lower PINP grade (*OR* = 0.91, 95% CI: 0.87–0.95, *P* < 0.001) (Table 4).

**TABLE 4:** Univariate ordinal logistic regression analysis.

Indicators	β	S. E	Wald	*P*-value	*OR* (95% CI)
Having fatty liver	2.30	0.52	19.56	<0.001	9.97 (3.59–27.69)
Age	0.35	0.11	10.12	0.001	1.43 (1.15–1.77)
BMI	0.15	0.03	25.00	<0.001	1.16 (1.08–1.24)
FINS	0.05	0.02	6.25	0.006	1.05 (1.02–1.09)
C-peptide	0.41	0.16	6.57	0.009	1.51 (1.11–2.07)
TG	0.95	0.39	5.93	0.015	2.58 (1.20–5.56)
AST	0.04	0.02	4.00	0.016	1.04 (1.01–1.07)
ALT	0.02	0.01	4.00	0.033	1.02 (1.01–1.03)
γ-GGT	0.05	0.02	6.25	0.019	1.05 (1.01–1.09)
HOMA-IR	0.23	0.08	8.27	0.006	1.26 (1.07–1.48)
LCAT	−0.10	0.02	25.00	<0.001	0.91 (0.87–0.95)

Multivariable ordinal logistic regression was performed to assess factors independently associated with lower PINP grade. In Model 1, which included MAFLD status, age, and BMI, MAFLD was significantly associated with lower PINP grade (*OR* = 5.76, 95% CI: 1.81–18.40, *P* = 0.003), and older age was also significantly associated with lower PINP grade (*OR* = 1.33, 95% CI: 1.06–1.68, *P* = 0.015). BMI was not independently associated with PINP grade (*OR* = 1.07, 95% CI: 0.99–1.16, *P* = 0.085). After further adjustment for LCAT in Model 2, MAFLD remained significantly associated with lower PINP grade (*OR* = 4.74, 95% CI: 1.41–15.99, *P* = 0.012), as did older age (*OR* = 1.38, 95% CI: 1.08–1.75, *P* = 0.009). In contrast, BMI (*OR* = 1.03, 95% CI: 0.92–1.15, *P* = 0.611) and LCAT (*OR* = 0.96, 95% CI: 0.89–1.03, *P* = 0.273) were not independently associated with PINP grade (Table 5).

**TABLE 5:** Multivariable ordinal logistic regression analysis.

Model	Indicators	β	S.E	Wald	*P*-value	*OR* (95% CI)
Model-1 (Not included in LCAT)	MAFLD	1.751	0.592	8.75	0.003	5.76 (1.81–18.40)
	Age	0.288	0.118	5.89	0.015	1.33 (1.06–1.68)
	BMI	0.070	0.041	2.97	0.085	1.07 (0.99–1.16)
Model-2 (Include LCAT)	MAFLD	1.557	0.620	6.31	0.012	4.74 (1.41–15.99)
	Age	0.321	0.123	6.83	0.009	1.38 (1.08–1.75)
	BMI	0.028	0.055	0.26	0.611	1.03 (0.92–1.15)
	LCAT	−0.044	0.040	1.20	0.273	0.96 (0.89–1.03)

Two models are presented: Model-1, excludes LCAT, while Model-2 includes LCAT., The table shows regression coefficients (β), standard errors (S.E.), wald statistics, p-values, and odds ratios (*OR*) with 95% confidence intervals (CI) for each independent variable.

Abbreviations: *OR*, odds ratio; CI, confidence interval.

The proportional-odds assumption was subsequently assessed using the Brant test. The omnibus test was nonsignificant for Model 1 (χ^2^ = 6.93, df = 6, *P* = 0.328) and Model 2 (χ^2^ = 9.19, df = 8, *P* = 0.327). In Model 2, the variable-specific tests were also nonsignificant for MAFLD status (*P* = 0.251), age (*P* = 0.376), BMI (*P* = 0.323), and LCAT (*P* = 0.403). These findings provided no evidence against the proportional-odds assumption for either multivariable model.

Additional covariate-adjustment analyses produced similar findings (Supplementary Table S1). After further adjustment for sex and Tanner stage, MAFLD remained associated with poorer PINP status (*OR* = 4.62, 95% CI: 1.36–15.75, *P* = 0.014). The association also remained significant after additional adjustment for 25-(OH)D and HOMA-IR (*OR* = 6.47, 95% CI: 1.54–27.24, *P* = 0.011). When sex, Tanner stage, 25-(OH)D, and HOMA-IR were simultaneously added to Model 2, the association persisted (*OR* = 6.35, 95% CI: 1.47–27.51, *P* = 0.013). These additional analyses showed no material attenuation of the association between MAFLD and poorer PINP status.

Multicollinearity was further assessed for Model 2. Although MAFLD status was strongly inversely correlated with serum LCAT (Spearman *r* = −0.713, *P* < 0.001), the VIF values for MAFLD status, age, BMI, and LCAT were 1.92, 1.17, 2.75, and 2.85, respectively. The corresponding tolerance values were 0.52, 0.86, 0.36, and 0.35, respectively, and the condition number was 3.40. These diagnostics did not indicate problematic multicollinearity in the final model. AST and ALT were also strongly correlated (*r* = 0.705, *P* < 0.001); however, they were not simultaneously included in the multivariable model.

Sensitivity analyses yielded results consistent with the primary analysis. In the mean bias-reduced adjacent-category ordinal regression model, MAFLD remained significantly associated with lower PINP status (β = −0.791, *P* = 0.030). Similarly, in the Firth penalized logistic regression model, MAFLD was significantly associated with PINP < P25 after adjustment for age, BMI, and LCAT (adjusted *OR* = 5.41, 95% CI: 1.65–19.82, *P* = 0.0048). The Monte Carlo Fisher-Freeman-Halton exact test further confirmed a significant difference in PINP-grade distribution among the three groups (*P* < 0.001). Collectively, these findings supported the robustness of the primary association despite the sparse distribution of PINP categories.

Sensitivity power analyses were performed for the available sample. For the 3 × 4 PINP-grade distribution, the observed effect size was Cohen’s ω = 0.51 (Cramér’s V = 0.36). With N = 127 and α = 0.05, the minimum detectable effect size at 80% power was ω = 0.33. For Model 2, the fixed-design simulation based on 5,000 replications yielded an estimated power of 72.8% for detecting the adjusted association between MAFLD and poorer PINP status. These findings suggested adequate sensitivity for detecting an overall group difference of the observed magnitude, but more limited sensitivity for the adjusted multivariable association.

## 4 Discussion

This study enrolled 42 control children, 41 with obesity-without-hepatic-steatosis, and 44 with MAFLD. Compared with controls, both obesity groups exhibited abnormalities in glucose and lipid metabolism parameters, indicating underlying metabolic dysregulation. Importantly, the obesity-without-hepatic-steatosis group should not be interpreted as representing metabolically healthy obesity, because metabolic disturbances, including insulin resistance, may still be present despite the absence of ultrasonographically detectable hepatic steatosis. Children with MAFLD demonstrated more pronounced metabolic disturbances, including altered liver function parameters, consistent with previous studies.

We observed differences in 25-hydroxyvitamin D and ALP levels across groups. The MAFLD group had significantly lower ALP values than the control group. Since ALP plays a crucial role in bone formation, its reduction may contribute to impaired bone metabolism (Tourkova et al., 2023). Furthermore, serum LCAT concentrations were lowest in the MAFLD group. This finding may be consistent with preclinical evidence indicating that dysregulation of the PP2Acα-LCAT pathway accompanies hepatic injury and that restoration of LCAT-dependent reverse cholesterol transport may contribute to improved bone homeostasis (Lu et al., 2022).

A key finding was that children with MAFLD had significantly lower PINP grades compared to both control and obese children, suggesting an association between MAFLD and abnormal bone turnover markers. While few studies have directly investigated bone turnover markers in pediatric MAFLD, existing evidence using bone mineral density (BMD) measurements has indicated a relationship between hepatic steatosis and bone control. Several studies have demonstrated that hepatic fat accumulation correlates with reduced BMD independent of obesity, highlighting the importance of osteoporosis prevention in children with MAFLD (Labayen et al., 2018; Chun et al., 2021; Labayen et al., 2023).

In the present study, we utilized established bone turnover markers (PINP and β-CTX) for early assessment of bone metabolism. This approach is particularly relevant in children, as BMD measurement in young populations presents technical challenges and reference data are limited (Madhuchani et al., 2023). Our results showed that children with obesity-without-hepatic-steatosis had lower PINP levels, higher β-CTX levels, and a lower PINP/β-CTX ratio compared to controls. Notably, the MAFLD group specifically showed significant alterations in PINP parameters but not in β-CTX, suggesting that MAFLD in children may primarily affect bone formation rather than bone resorption. This pattern could reflect the physiological predominance of bone formation during growth, although sample size limitations and disease heterogeneity warrant consideration (Pageau et al., 2025). Although chronological age did not differ significantly among groups, children with MAFLD were numerically older than those in the control and obesity without hepatic steatosis groups. Because bone turnover during childhood is strongly influenced by growth and maturation, this difference may have contributed to the observed variation in PINP status. We attempted to reduce this confounding by using age-, sex-, and puberty-specific PINP reference percentiles and by adjusting for age in the multivariable analyses. Nevertheless, some residual confounding related to developmental and metabolic factors could still remain. We therefore repeated the analysis with additional adjustment for sex, Tanner stage, 25-(OH)D, and HOMA-IR. The association between MAFLD and poorer PINP status remained significant across these models, suggesting that the observed relationship was not readily explained by differences in sex, pubertal stage, vitamin D status, or insulin resistance alone. However, the confidence intervals became relatively wide in the more extensively adjusted models, likely reflecting the modest sample size and sparse distribution of PINP categories. The magnitude of the association should therefore be interpreted with appropriate caution. The proportional-odds assumption was also not rejected for either primary multivariable model, providing further support for the use of the ordinal regression framework in the primary analysis.

In the univariable analysis, higher serum LCAT was associated with a lower likelihood of poorer PINP status, whereas this association was no longer statistically significant after multivariable adjustment. The partial correlation analyses showed a similar pattern. The relationship between LCAT and PINP grade persisted after adjustment for age and BMI but became weaker and nonsignificant once MAFLD status was additionally considered. Together with the strong inverse correlation between MAFLD status and LCAT, these findings suggest that the observed LCAT–PINP relationship may partly reflect metabolic information shared with MAFLD status. Importantly, the multicollinearity diagnostics did not indicate problematic collinearity, suggesting that the attenuation of the LCAT estimate in Model two is unlikely to be explained solely by numerical instability. However, neither partial correlation nor multivariable adjustment can establish mediation or causality in this cross-sectional setting. LCAT should therefore be regarded as a pathway-related biomarker associated with MAFLD and bone formation status rather than as an independently established determinant of impaired bone formation.

To our knowledge, this is the first clinical study to investigate both peripheral bone turnover markers and serum LCAT levels in children with MAFLD. It provides preliminary evidence linking reduced LCAT levels to impaired bone formation in this population. Several limitations should nevertheless be considered. The relatively modest sample size remains an important limitation, particularly for multivariable estimation. Although the sensitivity analysis indicated that the available sample could detect an overall PINP-grade difference of the observed magnitude, the simulation-based power for the adjusted association between MAFLD and PINP status was 72.8%, below the conventional 80% level. The sparse distribution of PINP categories further limited precision; in the MAFLD group, 39 of 44 children were classified as < P25, leaving only five participants across the remaining three categories. Together, the modest sample size and sparse outcome distribution likely contributed to the relatively wide confidence intervals of the multivariable estimates. Although the mean bias-reduced adjacent-category ordinal regression and Firth penalized logistic regression yielded findings consistent with the primary analysis, these methods cannot fully compensate for the limited information contained in sparsely populated outcome categories. The magnitude and precision of the adjusted association should therefore be interpreted cautiously and require confirmation in larger independent cohorts. Physical activity, dietary calcium and phosphate intake, pubertal growth velocity, and the PTH–calcium–phosphate axis were not directly assessed. Although PINP was standardized using age-, sex-, and puberty-specific reference data and children with conditions affecting bone metabolism were excluded, residual confounding by these unmeasured factors cannot be excluded. Although blood samples were generally collected under early-morning fasting conditions, residual diurnal variation in bone turnover markers, particularly β-CTX, cannot be completely excluded because the exact sampling time was not recorded. In addition, seasonal variation may have influenced serum 25-(OH) D concentrations because the sampling season was not formally accounted for. Hepatic steatosis was assessed by conventional ultrasonography, and quantitative MRI-PDFF was not available in the current cohort. Skeletal assessment was based on bone turnover markers without DXA measurements. These methodological limitations may have restricted quantitative characterization of hepatic fat burden and skeletal status. Moreover, no formal mediation analysis was performed. Therefore, the attenuation of the association between fatty liver status and PINP grade after inclusion of LCAT cannot be interpreted as evidence that LCAT mediates this relationship. Furthermore, the study design does not establish temporality between MAFLD, reduced LCAT, and impaired bone formation. Accordingly, the mechanistic role of LCAT remains hypothetical and requires validation in prospective and experimental studies. Future multicenter, prospective studies with larger cohorts and longitudinal designs are needed to validate these findings and elucidate the underlying causal mechanisms. Our center now has access to MRI-PDFF, and future prospective cohorts will incorporate quantitative hepatic fat assessment and, where available, pediatric DXA to further validate the observed association between MAFLD and bone formation.

## 5 Conclusion

MAFLD was independently associated with lower PINP grade after adjustment for the measured covariates. Reduced serum LCAT was associated with altered bone formation in children with MAFLD but was not an independent predictor after multivariable adjustment. It may therefore serve as a pathway-related biochemical marker, while its mediating or causal role remains unconfirmed. Longitudinal studies with formal mediation and mechanistic analyses are needed to clarify the MAFLD-LCAT-bone formation pathway and its potential relevance to early risk assessment and prevention in at-risk children.

## Data Availability

The raw data supporting the conclusions of this article will be made available by the authors, without undue reservation.
