# Supplementary material for: MAFLD and impaired bone formation in children: association with reduced serum LCAT
Source: Front Cell Dev Biol. 2026 Sep 25;14:1902360. doi: 10.3389/fcell.2026.1902360 (PMC13649763; doi:10.3389/fcell.2026.1902360)
Supplement: Supplementary file 1 [file Table1.docx]

**TABLE S1** Sensitivity analyses of the association between MAFLD and poorer PINP grade

| **Model** | **Additional covariates** | **OR for MAFLD** | **95% CI** | **P-value** |
| --- | --- | --- | --- | --- |
| Primary model | Age, BMI, LCAT | 4.74 | 1.41–15.99 | 0.012 |
| Model A | Primary model + sex + Tanner stage | 4.62 | 1.36–15.75 | 0.014 |
| Model B | Primary model + 25-(OH)D + HOMA-IR | 6.47 | 1.54–27.24 | 0.011 |
| Fully adjusted model | Primary model + sex + Tanner stage + 25-(OH)D + HOMA-IR | 6.35 | 1.47–27.51 | 0.013 |

**Notes:** ORs and 95% CIs were derived from ordinal logistic regression models, with higher ordinal scores representing poorer PINP status (lower PINP percentile categories). The primary model included MAFLD status, age, BMI, and LCAT. Model A additionally adjusted for sex and Tanner stage; Model B additionally adjusted for 25-(OH)D and HOMA-IR; and the fully adjusted model additionally included all four covariates.
